# Sexuality, sexually transmitted infections and contraception among health sciences students in university of Lomé, Togo

**DOI:** 10.1186/s13104-018-3923-3

**Published:** 2018-11-14

**Authors:** Tchin Darré, Bayaki Saka, Atchi Walla, Koumavi Didier Ekouévi, Koué Folligan

**Affiliations:** 1 Department of Pathology, University of Lomé, BP 1515, Lomé, Togo; 2 Department of Dermatology, University of Lomé, Lomé, Togo; 3 Orthopedic Trauma Department, University of Lomé, Lomé, Togo; 4 Department of Health and Epidemiology, University of Lomé, Lomé, Togo; 5 Department of Histology-Embryology, University of Lomé, Lomé, Togo

**Keywords:** Sexuality, Contraception, FSS-UL students, STI, Togo

## Abstract

**Objectives:**

Evaluate the practice of sexuality, contraception and the risk of sexually transmitted infections among students in the Faculty of Health Sciences, University of Lomé, Togo.

**Results:**

Three hundred and sixteen (316) students were interviewed, with a response rate of 43.3%. The average age of students completing the form was 21.4 ± 2.7 years and their sex ratio was 2.2. Of this number of students who completed the form, 51.8% have already had sex. The mean age of first intercourse was 17.9 ± 3.2 years; 70.3% were heterosexual. Regarding the number of sexual partners, 48.5% of students had more than one partner, of whom 15.9% had at least 5 sexual partners. 21.5% of these students had only one sexual intercourse per month. Regarding contraception among students with the card, 67.5% of students used a method of contraception. Among those using contraceptives, it was a 55.3% condom, followed by the Ogino method at 14.1%. Some of our respondents used more than one method of contraception and 28.5% of respondents indicated that their partners used a method of contraception. For STIs, 10.8% of students completing the form were already infected. Gonorrhea was reported in 30.4% of cases, candidiasis in 26.1% of cases.

## Introduction

The control and management of sexuality has been a subject of concern in recent years [1, 2]. In developing countries, sanitary priority is given to infectious, surgical, and nutritional pathologies [2]. Sexuality and contraceptive care are relegated to the second or third plane [3]. It must be noted that even in the faculties of health, this discrimination is in order.

In Togo, no study has been conducted on the subject of sexuality in both general population and young people in particular. Some questions can be asked including: how do students in the Faculty of Health Sciences manage their own sexuality? Do they receive the appropriate knowledge to manage their own sexuality or even manage and advise the population? The objective of this work was therefore to evaluate the practice of sexuality, contraception and the possible risk of sexually transmitted infection (STI) among health sciences students in university of Lomé.

## Main text

### Methodology

This was a descriptive cross-sectional study on the practices of sexuality and contraception among health sciences students in university of Lomé and their experience with STIs by the administering a questionnaire. Included were students regularly enrolled in the FSS during the 2017–2018 academic year and present at the time of the survey? Students in seventh and above were on hospital placements and were not included in this survey. The variables studied were: sociodemographic variables, the practice of sexuality and contraception of students and their experience with STIs.

### Results

Seven hundred and thirty (730) questionnaires were distributed but 316 returned to us duly completed, with a rate of 43.3%. There were 217 boys and 99 women, a sex ratio of 2.2. The average age of students was 21.4 ± 2.7 years (extremes: 16 and 28 years). First-year students were in the majority (25.6%). For marital status, 54.7% were single and 31.7% were in common-law. The notion of first sexual intercourse was filled by 293 students, 152 (51.8%) of whom said they had already had sex. The average age of first sexual intercourse was 17.9 ± 3.2 years with extremes of 16 and 24 years. The distribution of students according to their sexual practice showed a predominance of heterosexuality (70.3%) (Table 1). The majority of students (34.1%) had only one sexual partner (Table 1). The notion of contraception was found in 67.5% of students. It was condom in 55.3% of cases, followed by the Ogino method 14.1%. Some students used more than one contraceptive method (Table 1). The notion of emergency contraception was noted in 11.2% of students. STIs were reported in 10.8% of students. These were gonorrhea in 30.4% of cases, syphilis (17.4%), and hepatitis B (13.0%).

**Table 1:** Epidemiological and sexual characteristics of students

	Number (n)	Percentage (%)
Years of study (n = 316)		
1st	81	25.6
2th	26	8.3
3th	98	31.0
4th	18	5.7
5th	75	23.7
6th	18	5.7
Marital status (n = 316)		
Single	173	54.7
Free union	100	31.7
Not specified	43	13.6
Type of sexual practice (n = 219)^a^		
Heterosexuality	154	70.3
Masturbation	55	25.1
Homosexuality	5	2.3
Bisexuality	5	2.3
Number of sexual partners (n = 138)^b^		
No sexual partner	24	17.4
1 sexual partner	47	34.1
2 sexual partners	24	17.4
3 sexual partners	16	11.6
4 sexual partners	5	3.6
5 sexual partners	22	15.9
Type of contraception (n = 170)^c^		
Condom	94	55.3
Calendar method or Ogino	24	14.1
Coitus interrupted	23	13.5
Morning after pill	19	11.2
Oestro-progestative pills	5	2.9
Injections	3	1.8
Implants	2	1.2
Type of STI (n = 23)^d^		
Candida	6	26.1
Gonorrhea	7	30.5
Hepatitis B	3	13.0
Unlabeled sexual infection	3	13.0
Syphilis	4	17.4

^a^Only 219 students completed this item

^b^Only 138 students completed this item

^c^Only 170 students are sexually active

^d^23 students had STI

### Discussion

The filling rate of survey cards in our study was 43.3%. In Tunisia, Mseddi et al. had 44.0% of responses [4]. This low response rate in both studies can be explained by the taboo spirit that still surrounds sexuality in most African countries [2, 4]. Also, this was the first study of its kind in this faculty, which partly explains the low response rate. The majority of students (54.7%) were single, comparable to the results obtained in Bangui by Sepou with 55.2% of singles [5]. This can be explained by the fact that the majority of students would like to finish their studies before officially entering into a relationship. The average age of students at first sexual intercourse in our study is higher than that reported by Mabiala Babelaa in Congo which was 14.6 ± 1.7 years old [6]. Young people from the age of 18 often want to take the plunge and become adults through first sexual intercourse [6]. From then on, the student is attracted by this experience which he repressed because being minor [7]. The prevalence of heterosexual students (70.3%) observed in our series was also reported by Mseddi et al. in Tunisia in 60.1% of cases [4]. Our study reveals that 67.5% of students used a contraceptive and in most cases a Condom; followed by the Ogino method. In Congo in the series of Mabiala Babelaa et al., 42.2% of adolescents used a condom, 41.7% of adolescent girls used the Ogino method [6]. In our study, 28.5% of our respondents had revealed that their partners were using a contraceptive. Of these, 54.3% preferred the Condom. This corroborates the results in South Africa where Maharaj and Cleland found nearly 25.0% of young men and a third of young, heterosexual women who influenced their partners to use a Condom [8].

In the last 12 months, some 11.2% of our respondents had used emergency contraception. This may be due to lack of awareness of contraceptive methods, misuse of condoms, or occasional laxity in negligent contraceptive use [3, 9]. Of the students who responded to our questionnaire, 10.8% had had an STI. These were candidiasis in 26.1% of cases. Wasie et al. in Ethiopia had found a 6.4% history of sexually transmitted infections [10]. These results can be explained by the precocity and the non-protection during sexual intercourse [11].

Sexual conditions other than STIs were prevalent among students in 4.7% of cases. Of those who suffered 40.0% did not know what it was. This ignorance can be explained by the absence of a sexology class and the taboo surrounding sexuality in African society [12].

## Limitations

The relatively low response rate on the survey cards, because the study of the subject itself assesses the personal and sensitive problems related to sexuality. Furthermore, the statistical analysis did not find any risk factors of sexual practices, of having STIs, determinants of contraception. Finally, there were items that had not been completed by all 316 students.

## Abbreviation

STI: sexually transmitted infection
